# Multiple Defects in Muscle Regeneration in the HSALR Mouse Model of RNA Toxicity

**DOI:** 10.3390/ijms262210985

**Published:** 2025-11-13

**Authors:** Ramesh S. Yadava, Mira A. Zineddin, Mani S. Mahadevan

**Affiliations:** Department of Pathology, University of Virginia, Charlottesville, VA 22908, USA; zineddinmira@gmail.com

**Keywords:** muscle regeneration, myotonic dystrophy, satellite cells, RNA toxicity, fiber type, muscular dystrophy

## Abstract

Myotonic dystrophy type 1 (DM1) results from the toxicity of RNA produced from the mutant allele of the DMPK gene. The mechanism by which the toxic RNA causes muscular dystrophy in DM1 is unknown. Dystrophy in DM1 is associated with defective muscle regeneration and repair. Here, we used the BaCl_2_-induced damage model of muscle injury to study muscle regeneration in the HSALR mouse model of DM1. We have previously shown delayed muscle regeneration and deleterious effects on satellite cell numbers in another mouse model of RNA toxicity using similar experimental approaches. We found that HSALR mice show no apparent deleterious effects on satellite cell number or early markers of muscle regeneration. Further analysis at later time points after damage showed increased numbers of internal nuclei as compared to control mice undergoing the same protocol. Muscle fiber type analysis using immunostaining for type IIA and IIB fibers identified a switch to slower fibers (increased fraction of IIA and reduced fraction of IIB fibers) after regeneration in HSALR mice as compared to regenerated muscle from wildtype mice.

## 1. Introduction

Myotonic dystrophy type 1 (DM1), the most common muscular dystrophy in adults and children, affects skeletal muscles and many other organs in the body. DM1 is a genetic disorder that affects at least 1 in 8000 people worldwide, but recent studies suggest prevalence could be much higher [1,2]. A (CTG)n expansion in the 3′ untranslated region (3′UTR) of the dystrophia myotonica protein kinase (DMPK) gene results in DM1 [3,4]. The repeat expanded RNA (toxic RNA) accumulates as RNA foci in the nuclei [5,6]. The toxic RNA causes DM1 by adversely affecting RNA-binding proteins such as muscleblind-like family members (MBNL1–3) and CUGBP Elav-like family member 1 (CELF1). These RNA-binding proteins are involved in the control of alternative splicing of numerous transcripts in different tissues [7,8]. In addition, the toxic RNA also alters various downstream signaling pathways and proteins including glycogen synthase kinase 3b (GSK3b), AKT, AMPK, Staufen, hnRNPH and hnRNPF, DEAD-box protein (DDX6), RNA helicase p68/DDX5, TBPH (a homolog of human TAR DNA-binding protein 43 or TDP43), BSF (Bicoid stability factor), Fn14/TWEAK, and protein kinase C (PKC) [9,10,11,12,13,14,15,16,17,18,19]. Several mouse models that address the role of CUG repeats or the role of RNA-binding proteins targeted by CUG repeats have been used to understand the molecular basis of DM1 pathogenesis [20,21,22,23,24,25,26,27]. The HSALR mouse model is the most widely used for the role of CUG RNA in DM1 pathology; it expresses CUG repeats in the 3’UTR of the gene encoding human skeletal muscle actin [20]. These mice develop myotonia and skeletal muscle abnormalities, such as increased central nuclei and myofiber size variability, similar to the pathology seen in skeletal muscles of individuals with DM1. In addition, histological stains for succinate dehydrogenase and cytochrome oxidase show increased activity in vastus lateralis muscles from 6-month-old HSALR mice, a common feature of oxidative muscle fibers (type 1 fibers). In another study, HSALR mice at 6 months were also characterized by a reduction in fiber number in the gastrocnemius, but not in the tibialis anterior; this correlated with a reduction in grip strength [9].

Skeletal muscles fibers are distinguished by the expression of specific myosin heavy chains and are classified as MyHC-1/slow (MYH7), MyHC-2A(MYH2), MyHC-2X(MYH1), and MyHC-2B(MYH4). These fibers are present in mice, rats, and many other species, except that fibers expressing MYH4 are not present in most human muscles. The fibers differ in metabolism, with type 1 and 2A fibers being more oxidative and 2B fibers being more glycolytic. Type 1 fibers exhibit more endurance and are thought to function more in postural roles, whereas type 2 fibers are able to generate more rapid force (for strength, rapid movement, and shivering/thermostasis) but tire faster. Hybrid fibers containing a mix of two myosin heavy chains are also seen in muscles, often in response to exercise, variation in electrical stimuli, or pathology [28]. In mice, studies have examined the fiber type composition in the different muscles (soleus, extensor digitorum longus, tibialis anterior, and gastrocnemius). Aside from the soleus muscle which has a relatively equal proportion of fibers expressing MYH7 (type 1) and MYH2 (type 2A), most mouse muscles show a dearth of type 1 fibers. This has been attributed to different scales of size between mice and humans, as well as postural, speed of response, and thermostability needs. Thus, fiber distribution alterations in response to experimental conditions in mice may not parallel the changes seen with human muscle pathology.

Here, we studied muscle regeneration in response to damage in the HSALR model of RNA toxicity in DM1. We used the BaCl_2_-induced injury model of the tibialis anterior (TA) muscle. This approach has been standardized and used previously by many labs [29,30,31]. Recently, we used the same approach in the DM200 mouse model [32] and in mice deficient in MBNLs [33] to study the response to the damage. Our current study in HSALR mice shows evidence for fiber type switching in response to damage and shows qualitative differences between HSALR-, DM200-, and MBNL-deficient mouse models that may shed light on differences between mouse models of RNA toxicity and human variants of myotonic dystrophy (i.e., DM1 and DM2).

## 2. Results

### 2.1. HSALR Muscle Histology and Fiber Size Analysis

**Figure 1:**
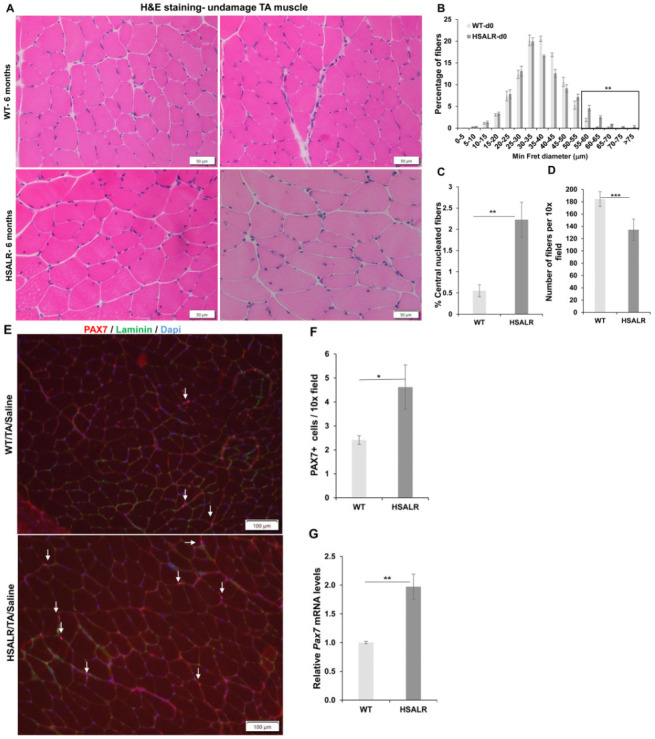
Variations in fiber size and MuSC number in the undamaged tibialis anterior (TA) muscles from 6-month-old HSALR mice. (**A**) A representative image of H&E staining of TA muscle from WT and HSALR 6-month-old mice. Scale bars = 50 μm. (**B**) Fiber size distribution shows an increase in the percentage of fibers, more than >55 μm (boxed area) in the non-injured TA of HSALR (fibers = 5050) as compared to WT mice (fibers = 5335) (*p* = 0.0016). *n* = 5 mice/group; ** *p* < 0.01. (**C**) Percentage of centrally nucleated fibers in TA from WT and HSALR mice. At least *n* = 5 mice were analyzed in each group; ** *p* < 0.01. (**D**) The average number of myofibers on transverse sections of (TA) muscles from 6-month-old HSALR and wildtype mice. *n* = 5 for HSALR (fibers = 4428) and *n* = 5 for wildtype (fibers = 5530); 10× field. At least six fields are counted for each mouse: *** *p* < 0.01. Scale bars = 100 μm. (**E**) Immunofluorescence for PAX7 (red) in uninjured TA muscle showing MuSCs in wildtype and HSALR mice; scale bar = 100 μm. Nuclei were stained with DAPI (blue). Arrows represent PAX7+ cells. (**F**) Quantification shows an increase in PAX7+ cells (MuSCs) per 10× field in TA of HSALR compared to wildtype mice. *n* = 5 mice/group; at least nine non-overlapping sections/mouse; * *p* < 0.01. (**G**) Quantitative RT-PCR shows increased expression of *Pax7* in skeletal muscle (TA) of HSALR mice at six months old. The result from wildtype mice is set at a level of 1.0 for graphs in F and G. *n* = 5 mice/group; ** *p* < 0.01; Student’s *t*-test; Error bars are mean ± SEM. Abbreviations, TA—tibialis anterior; WT—wildtype.

A previous study indicated a decrease in the grip strength of HSALR mice from 3-month-old and 6-month-old mice compared to control mice of the same age [9]. In addition, the total number of myofibers per field in TA muscles in 6-month-old mice was also reduced compared to wildtype mice of the same age in the same study [9]. We analyzed grip strength in HSALR mice at 2 months and 6 months of age. Similar to the previous reports, we also found a decrease in the grip strength in 6-month-old HSALR mice as compared to the grip strength in the same mice at two months of age (*p* = 0.00039) (Figure S1). Thus, we chose to do further studies in 6-month-old HSALR mice. First, we evaluated the histology of undamaged TA muscles from HSALR and wildtype mice (FVB) (Figure 1). H&E-stained sections showed increased muscle fiber size variation and central nuclei (Figure 1A). The fiber size distribution analysis of TA muscles from HSALR mice showed a significant increase in the percentage of larger fibers (>55 μm diameter), 8.34% (HSALR) vs. 2.14% (WT) (*p* = 0.0029) (shown in the box area, Figure 1B). HSALR mice showed clear evidence of pathology with an increased rate of fibers with central nuclei (2.22% of fibers in HSALR as compared to 0.6% in wildtype) (*p* = 0.0051) (Figure 1C), though this is much less than what is typically found in biopsies from DM1 individuals [34,35]. We also counted myofibers on the transverse sections of tibialis anterior (TA) muscles from 6-month-old HSALR and wildtype mice. We found that the total number of myofibers was reduced significantly (*p* = 0.00075) in the HSALR mice compared to wildtype mice (Figure 1D). Next, we evaluated the number of satellite cells (also called muscle stem cells; MuSCs) in the TA muscles of HSALR mice using immunofluorescence (IF) for PAX7 as a marker for MuSCs. We found a significant increase (*p* = 0.047) in the number of MuSCs in HSALR TA muscles as compared to wildtype mice (Figure 1E,F). Similarly, using qRT-PCR, we found a substantial increase in *Pax7* mRNA levels (*p* = 0.004) in HSALR mice (Figure 1G). We also analyzed the satellite cells by examining the colocalization of PAX7-IF and MyoD-IF to determine whether the MuSCs are in a resting (Myod-ve) or activated state (MyoD+ve). Surprisingly, we found a higher proportion of double-positive satellite cells in the HSALR mice compared to wildtype mice (36 of 132 MuSCs in HSALR vs. 15 of 119 in WT mice). These results suggest that more satellite cells in the HSALR mice are in the activated state and ready to differentiate into myoblasts.

### 2.2. Muscle Regeneration Markers Are Similar in Wildtype and HSALR Mice

**Figure 2:**
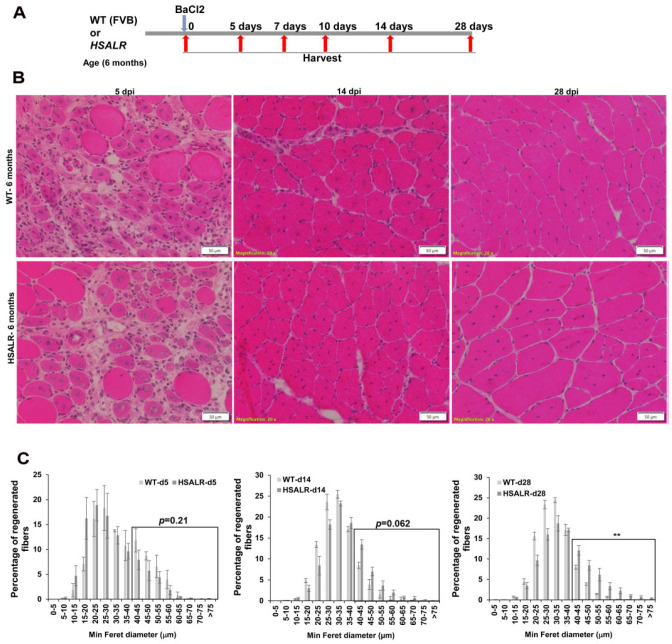
Muscle regeneration is similar in HSALR mice and wildtype mice. (**A**) Schematic showing the experimental design used in this study. (**B**) Representative images of H&E-stained TA muscle sections 5, 14, and 28 days post injury (dpi) in the WT and HSALR mice. *n* = 3–5 mice per group; scale bars = 50 μm. (**C**) Fiber size distribution analysis at 5, 14, and 28 days post injury (dpi) between WT and HSALR mice. Boxed area used for analysis shows gradual increase in fiber size in HSALR. *n* = 3–5 mice per group; ** *p* < 0.01; Student’s *t*-test; *p*-values are indicated on the graphs; error bars are as mean ± SEM.

We utilized an induced damage protocol using barium chloride (BaCl_2_) injections into the TA muscles of HSALR mice to study the effects of expanded (CUG) RNAs on muscle regeneration [30]. We used a cohort of 6 month old male and female HSALR mice. All mice had myotonia, as confirmed by electromyography (EMG). We injected 50 ul of a 1.2% BaCl_2_ solution into multiple regions of the right TA of each mouse and an equivalent volume of PBS into the left TA as an undamaged control. We also did the same experiment in wildtype (FVB) mice (6 months old) as a control. The details of this protocol are represented in Figure 2A. We collected tissues from the wildtype and HSALR groups at various times post injury (0, 5, 7, 10, 14, and 28 days). We used tissue staining, immunofluorescence, and quantitative RT-PCR to analyze histology, satellite cells, and muscle regeneration markers. We first studied the histology of the TA muscles at 5 days post injury. We focused on the area where damage was evident, based on the presence of fibers with central nuclei. In HSALR mice, at 5 days post injury (dpi), H&E staining revealed evidence of regeneration, with the number of centrally nucleated fibers being similar to that in wildtype mice (Figure 2B). The staining also showed that fiber maturation and repair of muscle morphology were well underway by 14 days and 28 days post injury. However, the number of internal nuclei in HSALR mice was increased compared to wildtype mice (Figure 2B). The fiber size distribution showed that damaged TA muscles in HSALR mice at 28 days post injury showed a significantly (*p* = 0.0094) increased proportion of larger fibers compared to wildtype mice. This was similar to what we observed in undamaged TA muscle from HSALR mice. However, there were no differences in fiber size distribution compared to wildtype mice at d5 (*p* = 0.20) and d14 (*p* = 0.062) post injury (Figure 2C), though it was trending towards the changes noted at 28 days post injury.

**Figure 3:**
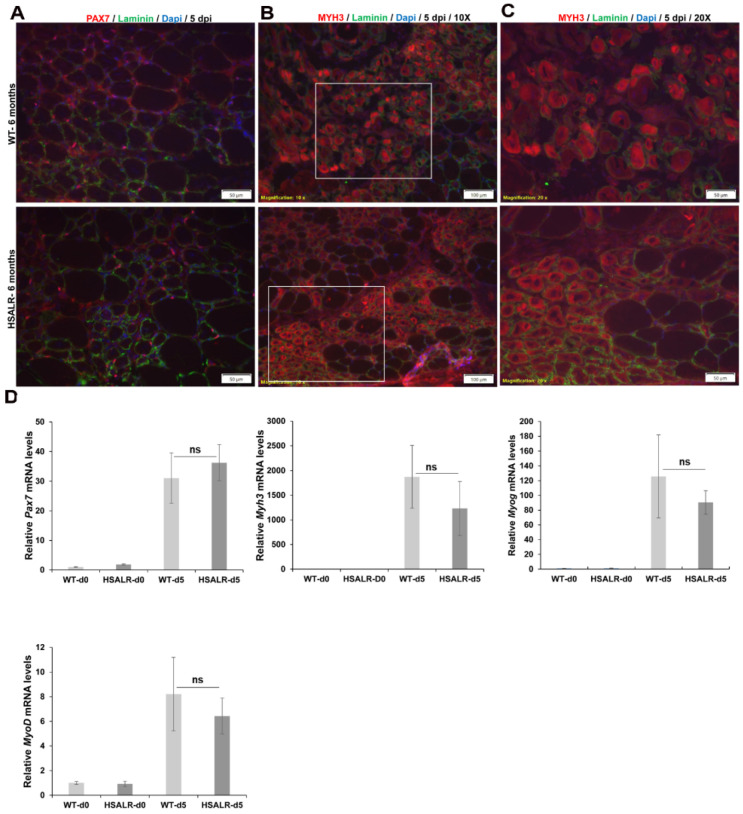
Satellite cell proliferation and muscle regeneration markers are not affected in HSALR mice. (**A**) Immunofluorescence of PAX7 (red, 50 μm scale), (**B**) MYH3 (red, regeneration marker, 100 μm scale)**,** and (**C**) MYH3 (red, regeneration marker, 50 μm scale) at higher magnification from the boxed area in (**B**) at five days post injury shows similar extent of muscle regeneration in HSALR compared to wildtype mice. Nuclei are stained with DAPI (blue) and laminin IF to outline muscle fibers (green). (**D**) Quantitative RT-PCR also shows no significant change in the expression of *Pax7*, *Myh3*, *Myod*, and *Myog* mRNA in skeletal muscle (TA) of HSALR mice compared to wildtype mice at 5 days post injury. ns-not significant. *n* = 5 mice per group; error bars are as mean ± SEM; light gray bars represent WT and dark gray bars represent HSALR.

Next, we used immunofluorescence for PAX7 (a marker of MuSCs) to determine changes in MuSC numbers, and immunofluorescence for MYH3 (embryonic myosin heavy chain, a marker of nascent myofiber formation) to determine the extent of muscle regeneration at 5 days post injury. PAX7-IF on TA muscles from HSALR mice at 5 days post injury revealed a robust increase in PAX7+ve cells in response to damage, similar to wildtype mice (Figure 3A). Also, MYH3-IF showed similar staining in HSALR mice as compared to wildtype mice at 5 days post injury, indicating no obvious difference in the extent of early muscle regeneration. (Figure 3B,C). We also did quantitative RT-PCR of *Pax7*, *Myh3*, *Myog*, and *MyoD* mRNA on RNA extracts from TA muscles collected 5 days post injury (Figure 3D). We found no significant change in the expression of all these muscle regeneration markers at 5 days post injury in HSALR mice compared to wildtype mice. Also, we did not notice a change in most of the regeneration markers except for Pax7 in undamaged TA muscle of HSALR and wildtype mice (Figure 3D, d0 data). These analyses are consistent with the conclusion that RNA toxicity in HSALR mice does not adversely affect satellite cell activation and muscle regeneration at 5 days post injury.

### 2.3. Histopathological Defects in Regenerated TA Muscles from HSALR Mice

**Figure 4:**
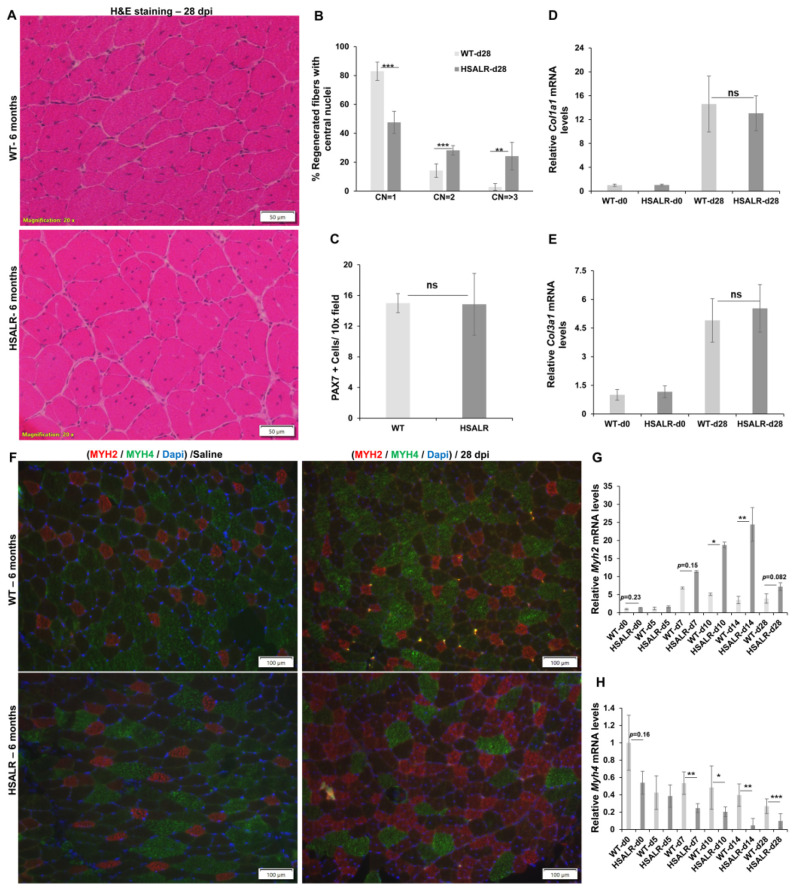
TA muscle in HSALR at 28 days post injury. (**A**) Representative image of H&E-stained TA muscle sections 28 days post injury (dpi). Scale bars =Scale bars = 50 μm. (**B**) The percentage of regenerated fibers with central nuclei (CN = 1 (*p* = 4.5507 × 10^−5^), CN = 2 (*p* = 0.000563), and CN ≥ 3 (*p* = 0.0012845)). *n* = 5 mice per group; *n* = 1100 fibers for WT and 1206 fibers for HSALR. (**C**) Quantification shows a similar level of MuSCs per 10× field in TA of HSALR compared to WT mice at 28 days post injury. *n* = 5 mice/group. At least five fields are counted per mouse. (**D**,**E**) Quantitative RT-PCR also shows no significant change in the expression of *Col1a1* and *Col3a1* mRNA in TA of HSALR mice compared to wildtype mice at 28 days post injury. ns-not significant. *n* = 5 mice per group; Student’s *t*-test; error bars are mean ± SEM. (**F**) MYH2 IF (Red) and MYH4 IF (green) in TA muscle sections 28 days post injury (BaCl_2_) show an increased number of MYH2 fibers in HSALR mice compared to WT mice. There was no significant difference in the expression of MYH2 and MYH4 in non-injured muscle (saline) from the same HSALR and WT mice. Nuclei are stained with DAPI (blue). Scale bars = 100 μm. (**G**) Quantitative RT-PCR shows a significant increase in *Myh2* mRNA expression in TA of HSALR mice during regeneration (0–28 days post injury) compared to wildtype mice. (**H**) Quantitative RT-PCR shows a significant decrease in *Myh4* mRNA expression in skeletal muscle (TA) of HSALR mice during regeneration (0–28 days post injury) compared to wildtype mice. ns-not significant. *n* = 5 mice per group; * *p* < 0.05, ** *p* < 0.01; *** *p* < 0.001, Student’s *t*-test; error bars are mean ± SEM.

Next, we assessed histological differences at a later time point. Usually, the histological appearance of wildtype skeletal muscle 28 days post injury shows that most regenerated fibers exhibit central nuclei and muscle structure more closely resembles non-damaged wildtype skeletal muscle, as shown in Figure 1A. The tissue sections of HSALR mice 28 days post injury have intact fibers, but with many more central nuclei (CN) compared to wildtype mice (Figure 4A). In induced damage experiments like the ones used in this study, regenerated fibers are characterized by the presence of central nuclei. We counted the number of regenerated fibers with one, two, or three or more central nuclei in wildtype (fibers = 1100) and HSALR (fibers = 1206) mice. The fibers in HSALR mice at 28 days post injury had a significantly increased percentage with more than one central nuclei (for CN = 2; 52.4% vs. 17.1%; (*p* = 0.000046); for CN = >3; 24.2% vs. 2.9%, *p* = 0.0013) compared to wildtype mice (Figure 4B). Thus, WT mice had about 80% of regenerated fibers with only one CN, whereas HSALR mice had 76% of regenerated fibers with more than one CN.

We also counted the number of MuSCs in HSALR mice at 28 days post injury and found no significant difference in the number of PAX7+ve cells in HSALR mice compared to wildtype mice (Figure 4C). We used qRT-PCR to assess the expression of two collagen genes, *Col1a1* and *Col3a1*, two markers of increased fibrotic activity. We found no evidence of increased expression of the collagen genes in HSALR mice compared to wildtype mice at 28 days post injury (Figure 4D,E).

Next, we analyzed the expression of MHY2 (type IIA) and MYH4 (type IIB) in the TA muscle collected at 28 days post injury. Usually, in mature murine TA muscle, the fibers expressing MYH2 (type IIA) are a minority, and fibers with MYH4 (type IIB) are the majority (Figure 4F). MYH7 (type I) is not notably expressed in TA muscle [36]. Immunofluorescence analysis for MYH2 and MYH4 showed that TA muscles from wildtype and HSALR mice injected with PBS had similar proportions of fibers expressing the respective proteins (Figure 4F). This showed that RNA toxicity had no noticeable effect on the proportion of type IIA and type IIB in undamaged TA muscles. However, in the BaCl_2_-damaged TA muscles, HSALR mice showed fewer MYH4 fibers and increased MYH2 fibers compared to the wildtype muscle (Figure 4F). Representative images at lower magnifications show clear evidence of fiber type transition (type IIB to type IIA) in HSALR mice at 28 days post injury (Figure S2). We also performed quantitative RT-PCR of *Myh2* and *Myh4* mRNA expression in TA muscles from wildtype and HSALR mice at 0, 5, 7, 10, 14, and 28 days post -injury (Figure 4G,H). In WT mice, there was a transient increase in *Myh2* expression by day 7 (approximately a five-fold increase) that was associated with an approximately 50% decrease in *Myh4* expression. At 10 days post injury and later time points, the level of *Myh2* expression decreased to about 3–4 times compared to baseline levels, whereas *Myh4* expression stayed at between 30 and 50% of baseline. In contrast, in the HSALR mice, *Myh2* expression was about 11-fold higher than baseline at 7 days post injury and continued to rise to about 20-fold higher by 14 days post injury (a point when myofiber identity is well established). This was accompanied by a progressive decrease in *Myh4* expression such that at 14 days post injury, *Myh4* expression was approximately 20-fold less than baseline levels (Figure 4G,H). This was consistent with the differences in fiber type distribution seen by IF. These data demonstrate significant differences in the fiber type distribution, after repair in the damaged TA muscle of HSALR mice as compared to wildtype mice.

### 2.4. Study of HSALR Mice at 2 Months of Age

**Figure 5:**
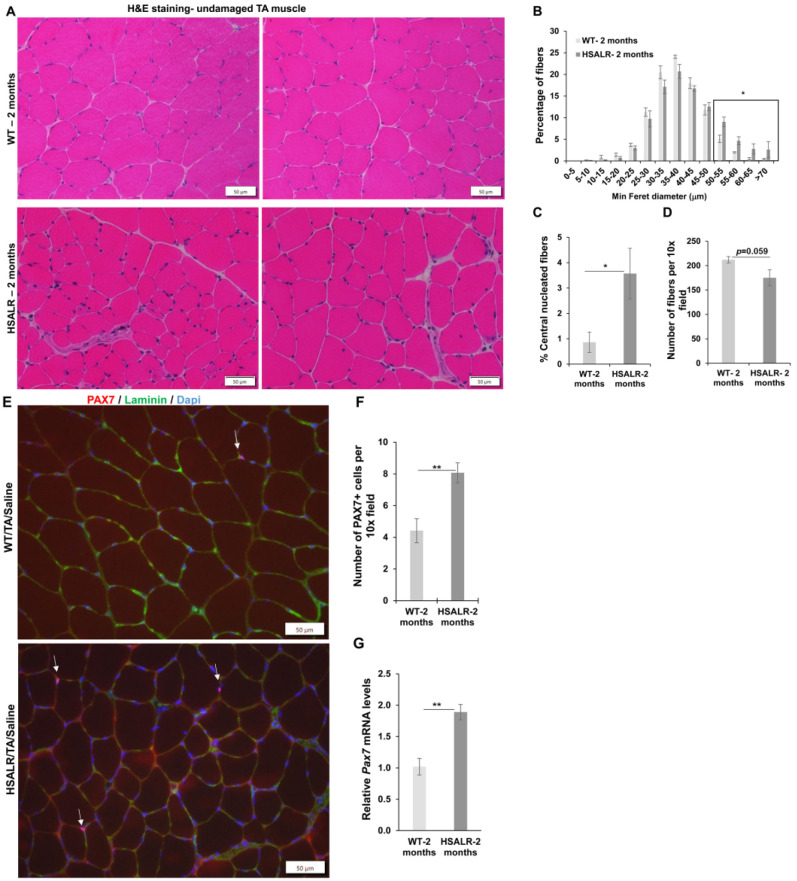
Characterization of tibialis anterior (TA) of 2-month-old HSALR mice. (**A**) A representative image of H&E-stained TA muscles of non-injured muscle from WT and HSALR 2-month-old mice. Scale bars = 50 μm. (**B**) Fiber size distribution shows an increase in the percentage of fibers (boxed area, >50 μm) in the non-injured muscle of HSALR compared to wildtype mice (*p* = 0.02). *n* = 4 for HSALR and *n* = 5; 10× fields used for analysis. * *p* < 0.05. (**C**) Centrally nucleated fiber percentage in sections of uninjured TA from HSALR mice are significantly (*p* = 0.026) increased compared to WT mice. At least *n* = 5 mice were analyzed in each group; * *p* < 0.05. (**D**) Average number of myofibers on transverse sections of TA muscles from 2-month-old HSALR and WT mice. *n* = 4 for HSALR (fibers = 1776) and *n* = 5 WT (fibers = 2119) in 10× field. *p* = 0.059. (**E**) PAX7 (red) Immunofluorescence of TA showed increased numbers of MuSCs (white arrows) in HSALR compared to WT mice. Nuclei are stained with DAPI (blue) and laminin IF was used to outline muscle fibers (green). Scale bars = 50 μm. (**F**) Quantification shows an increase in MuSCs per 10x field in the TA of HSALR compared to WT mice. *n* = 3–4 mice/group, at least seven non-overlapping sections/mouse; ** *p* < 0.01; Student’s *t*-test; error bars are mean ± SEM. (**G**) qRT-PCR shows increased expression of *Pax7* mRNA levels in the TA of HSALR mice as compared to WT mice. Muscles from at least four mice per group; ** *p* < 0.01; Student’s *t*-test; error bars are mean ± SEM.

We wanted to determine if the age of HSALR mice has any effect on muscle regeneration response to damage. We used 2-month-old HSALR mice similar to our previous studies of the DM200 mouse model and Mbnl1 knockout mice. We compared two-month-old HSALR to age-matched wildtype (FVB) mice. H&E-stained sections of undamaged TA muscle showed that HSALR mice had larger fibers, more fibers with central nuclei, and fiber size variations compared to wildtype mice (Figure 5A). These results are similar to what we observed in 6-month-old TA muscles. Next, we analyzed the fiber size distribution. We found that TA muscles from HSALR mice showed a significant increase in the percentage of fibers (>50 µm) (*p* = 0.037) and a substantial increase in the fibers with central nuclei (*p* = 0.026) (Figure 5B,C). We also found a decrease in the number of fibers in HSALR mice (*p* = 0.059) (Figure 5D). In 6-month-old mice, the lower number of fibers was even more evident as compared to WT mice (*p* = 0.00075) (Figure 1D). We then assessed the number of MuSCs and *Pax7* expression in the TA muscles of 2-month-old mice. Using PAX7 IF, we found a significant increase in the number of MuSCs in HSALR TA muscle compared to wildtype mice (*p* = 0.00997) (Figure 5E,F). Similar increases were seen using qRT-PCR for *Pax7* (*p* = 0.0094) (Figure 5G). The fold increase in the number of MuSCs and *Pax7* mRNA were similar to that seen with 6-month-old HSALR mice.

**Figure 6:**
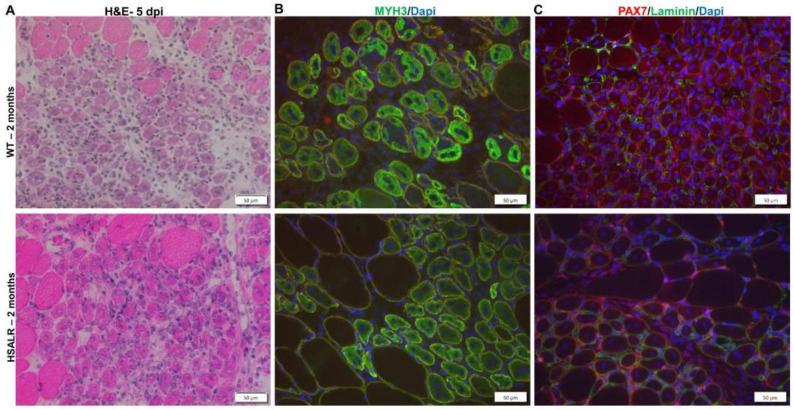
Early muscle regeneration markers in 2-month-old HSALR mice are similar to WT mice. (**A**) Representative images of H&E-stained muscle sections, (**B**) MHY3-IF, and (**C**) PAX7-IF show similar levels of muscle regeneration in HSALR mice compared to wildtype mice at 5 days post injury (5 dpi). Scale bars = 50 μm.

Next, we analyzed muscle regeneration response to damage at 5 days post injury (dpi) using H&E staining. We noted consistent evidence of regeneration with many small fibers with central nuclei, similar to the observations we made in 6-month-old mice. We assessed muscle regeneration using MYH3-IF and found a similar extent of regeneration in HSALR and wildtype mice. We also assessed satellite cell activation using PAX7-IF. We found no defect in MuSC activation in HSALR as compared to WT mice (Figure 6A–C).

**Figure 7:**
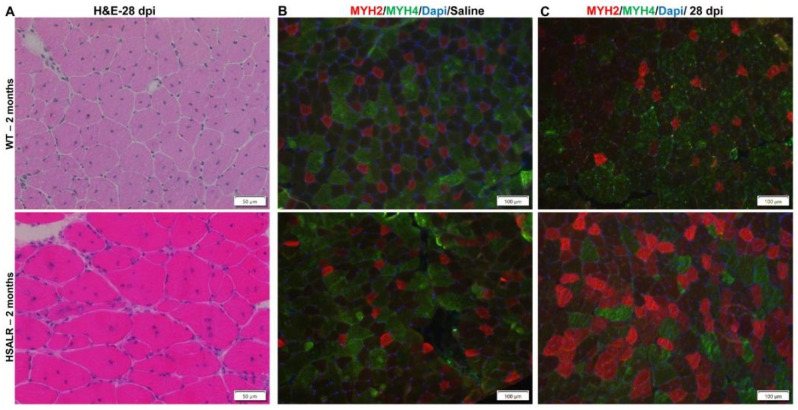
HSALR muscle shows fiber type switching at 28 days post injury in 2-month-old mice. (**A**) Representative image of H&E-stained TA muscle sections at 28 days post injury (dpi). Scale bars are shown. (**B**,**C**) MYH2 IF (Red), and MYH4 IF (green) in TA sections from uninjured TA (saline) (**B**) and 28 days post injury (28 dpi). (**C**) show an increased proportion of MYH2 fibers in the regenerated TA muscles of HSALR mice as compared to WT mice. Nuclei are stained with DAPI (blue). Scale bars are shown.

Further analyses at 28 days post injury showed similar findings to those in the 6-month-old mice. H&E staining showed increased multiple internal nuclei in HSALR mice compared to wildtype mice (Figure 7A). The immunofluorescence analysis for MYH2 and MYH4 showed that the TA muscles of WT and HSALR mice injected with PBS have similar proportions of fibers expressing each protein (Figure 7B). In contrast, the TA muscle from HSALR mice showed fewer MYH4 fibers (green) and an increased proportion of MYH2 (red) fibers as compared to the wildtype muscle at 28 days post injury (Figure 7C). All these data indicate that the duration of RNA toxicity (2 vs. 6 months) was not a major factor affecting muscle regeneration in response to damage, at least within the time frame of this study. However, we saw mild age-related changes. Though the number of MuSCs and *Pax7* mRNA levels were similar, there was a small change (decrease) in the number of fibers in the 10× field at 6 months old, compared to results in the 2-month-old HSALR mice. These data correlated with a significant drop in grip strength in the 6-month-old HSALR mice (Figure S1).

Since their first discovery, satellite cells (MuSCs) have been studied for their role in muscle maintenance and repair [37]. MuSCs are usually quiescent in the adult skeletal muscle and are responsible for muscle regeneration in response to injury. PAX7 is expressed in quiescent and activated MuSCs [38]. There are minimal studies on the number of satellite cells and their status in DM1. In one study, a loss of satellite cells was reported in muscle from children with congenital DM1 [39]. In contrast, an increase in satellite cell number in the distal TA muscles compared to the proximal VL muscle has been reported [40]. We observed an increase in satellite cell number due to RNA toxicity in the undamaged TA muscles of HSALR mice as early as 2 months of age, and this increase persisted at 6 months of age, suggesting that the number of MuSCs was elevated in the early stages of the disease. This increase in satellite cell number was not associated with enhanced early muscle regeneration after induced damage in HSALR mice, irrespective of age, unlike with MBNL1 overexpression, which led to increased numbers of MuSCs and a faster regenerative response [33,41]. In both cases, a fraction of satellite cells was in an activated state (Pax7^+^/MyoD^+^) and potentially committed to differentiation. Of note, the increase in MuSC numbers in HSALR mice was not affected, even after reducing levels of the toxic RNA by 90% with an antisense oligonucleotide (ASO) targeting the HSALR transcript (Figure S3). Thus, the increase in MuSCs seems unrelated to the toxic RNA, and the result suggests an alternate, unknown mechanism for the increase in HSALR mice. However, in the DM200 mouse model, the decrease in satellite cell numbers improved after using an ASO to reduce the level of toxic RNA [32]. This showed a clear link between the toxic RNA and its deleterious effects on MuSCs in the DM200 model.

## 3. Discussion

**Figure 8:**
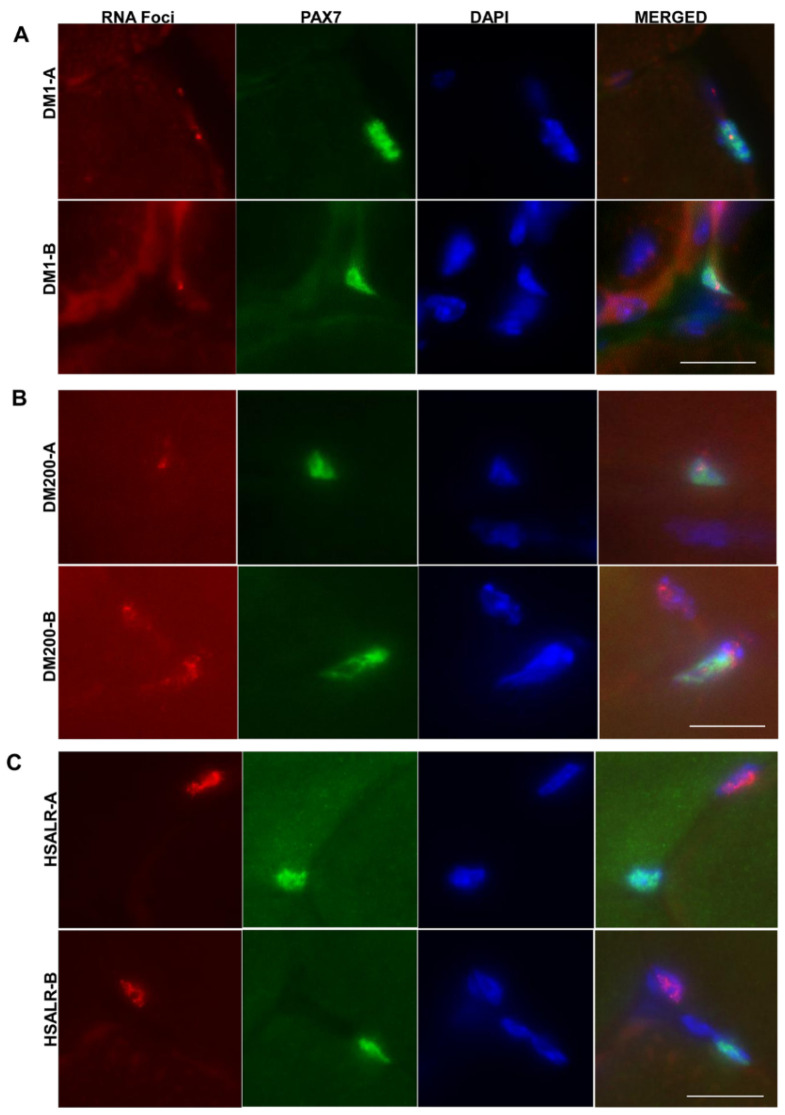
RNA foci in satellite cells. (**A**) PAX7 IF (green) and RNA FISH (red dots) in skeletal muscle from DM1 patients show PAX7+ve cells with RNA foci. (**B**) PAX7 IF (green) and RNA FISH (red dots) of the TA of a DM200 D+ mouse showing PAX7+ve cells with RNA foci. (**C**) PAX7 IF (green) and RNA FISH (red dots) of the TA muscle of a HSALR mouse showing PAX7+ve cells with no RNA foci. Nuclei are stained with DAPI (blue). Scale bar = 5 µm.

This study focused on an experimental approach to induce skeletal muscle damage and investigate the regenerative response in the HSALR mouse model of RNA toxicity in DM1. We have recently used similar methods in studying the regenerative response in the DM200 model of RNA toxicity [32] and mouse models of MBNL deficiency [33]. In the current study, we found that in the tibialis anterior (TA) muscle of HSALR (1) satellite cells (MuSCs) are increased, (2) early regenerative response to damage is similar to the wildtype, and (3) there are alterations in repair in the latter parts of muscle regeneration. However, this contrasts with a similar study we published on the DM200 mouse, where RNA toxicity results in fewer MuSCs, which delays the regenerative response. These differences in response to damage could be due to differences in the expression of repeat RNAs in MuSCs. In the HSALR mice, the transgene is driven by the human skeletal actin (HSA) regulatory regions. HAS is a gene that is only expressed in differentiated muscle [20]. In contrast, in the DM200 mouse model, the transgene is expressed using the human DMPK promoter, which is known to be expressed in some fraction of satellite cells. Using RNA-FISH, we found no evidence of RNA foci in the MuSCs of HSALR TA muscle. Given the promoter used to drive the HSALR transgene, it was not surprising to find no RNA foci in MuSCs; we evaluated over 100 satellite cells from multiple sections from different HSALR mice. But in the DM200 TA muscle and muscles from individuals with DM1, some portion of the population of MuSCs express mutant *DMPK* mRNAs (Figure 8, [32]).

One of the interesting observations made in this study was that regenerated fibers in HSALR mice have multiple central nuclei after 28 days post injury, in contrast to wildtype mice (Figure 5). Whereas, in the DM200 mouse model, regenerated fibers have mostly one nucleus after 28 days post injury [32], similar to wildtype mice. During muscle regeneration after necrotic injury (as happens with BaCL_2_ injection), regenerating myofibers are formed after fusion of myoblasts that are derived from the expansion of the MuSC pool, and the subsequent differentiation of activated MuSCs. This accumulation and division of MuSC-derived cells occurs in the first 4–7 days after damage [42,43,44]. Very few nuclei are added after that. The end result is a tremendous accretion of nuclei which are positioned centrally in long nuclear chains in the regenerating fibers [42,43,45,46]. In mice, these central nuclei remain post injury for many months after the fiber has been regenerated [42,43,47,48].

Why do a higher proportion of regenerated fibers in HSALR mice have multiple central nuclei in comparison to the DM200 model or wildtype mice? Given that the central nuclei in regenerated fibers are a result of the addition of new nuclei from fused myotubes (see review [42]), this may be related to the fact that HSALR mice have more MuSCs and more of these are activated (i.e., PAX7^+^/MyoD^+)^. In response to damage, this would result in more myoblasts being available for myotube formation and subsequent incorporation into regenerating fibers.

The accretion and positioning of nuclei are distinctly different in muscle repair due to chronic situations and physiologic hypertrophy from exercise, where focal damage or localized demands for repair are necessary, as opposed to the wholesale synthesis of new fibers (see reviews [42,43]). In such situations, in normal muscle, the MuSC response is not as massive, is more localized, and results in the addition of a few nuclei that are peripherally located on the myofiber. However, there are several muscle diseases characterized by abundant central nuclei such as myotubular myopathy and other rare centronuclear myopathies [49], where the loss of or severely reduced levels of specific proteins leads to the disease. This is usually associated with a distinct lack of evidence for active muscle regeneration. The central nuclei muscle pathology in DM1 resembles these other diseases, in that muscle pathology in DM1 is usually associated with a dearth of regeneration markers. This is distinct from the central nuclei in muscle pathology from Duchenne muscular dystrophy and associated disorders, where a loss of muscle membrane integrity leads to repeated cycles of necrosis and regeneration, which results in central nuclear chains.

We have very little knowledge about the mechanisms of nuclear positioning in DM1. Some of the genes involved in centronuclear myopathies (*BIN1* and *RYR1*) also exhibit alternative splicing defects in the skeletal muscles of individuals affected by DM1 [50,51]. For instance, aberrant alternative splicing resulting in the increased exclusion of a muscle-specific exon (exon 11) in *BIN1* has been reported [50]. This group went on to show that the gene therapy-mediated exclusion of *Bin 1* exon 11 in wildtype mice resulted in decreased strength. Subsequently, another group reported identifying a mutation at the 3′ splice acceptor site of intron 10 of *BIN1* that resulted in an autosomal recessive form of a rapidly progressive and lethal myopathy in affected children [52]. This group also identified a similar mutation in the canine inherited myopathy of the Great Dane dog model, which had a later onset but rapidly progressive phenotype as well. In both situations there was almost complete exclusion of exon 11+ isoforms, and abundant central nuclei on muscle histology. They subsequently made a mouse model that completely excluded exon 11 and showed that the constitutive or induced loss of this isoform had little effect on muscle strength, function, or phenotype except for smaller fibers after induced damage [53]. Thus, the mouse model differs from the human condition. Another relevant issue is that adult DM1 tissues show mostly exon 11+ isoform expression with perhaps 10–30% being the exon 11-ve isoform; they do not show a total absence of the exon 11+ isoform (Figure S4). Of note is the fact that the heterozygous parents of the probands in both the human and canine situations were completely asymptomatic [52] despite presumably expressing equal levels of exon 11+ and exon 11-ve isoforms, which brings into question the relevance of this splicing event to DM1-associated phenotypes. Nevertheless, as guided by the reviewer’s queries, we evaluated the splicing of *Bin1* exon 11 in the TA muscles of HSALR mice. We did find evidence for a minor increase in the exon 11-ve isoform, but this did not change over the course of regeneration (Figure S4). Thus it is difficult to attribute the altered splicing of *Bin1* exon 11 as a cause of the increased presence of central nuclei in regenerated HSALR TA muscles.

Several studies have also explored the expression and splicing of nuclear envelope transmembrane proteins, specifically SYNE1, in DM1 [54,55]. A SYNE1 isoform, which differs by 69 nucelotides (also called exon DV23), is differentially spliced in fetal and adult human skeletal muscle [56]. In skeletal muscles of individuals with DM1, there is an increase in the fetal isoform (Figure S5A) consistent with the overall transition from adult to fetal splice isoforms that occurs in DM1. Additionally, in another study, *Syne1* knockout mice exhibit defects in nuclear positioning in their muscle fibers [57]. We evaluated *Syne1* splicing in the HSALR and DM200 mice and found increased expression of the fetal isoform in the TA muscles of both models (Figure S5B). However, we did not find any correlative differences in splicing defects in *Syne1* and centronuclei in HSALR and DM200 mice when comparing non-damaged and damaged skeletal muscles (Figure S5). We have previously reported on the effect of MBNL loss on skeletal muscle regeneration [33]. Upon revisiting the study, we noted multiple internal nuclei in the myofibers of the regenerated TA muscles of *Mbnl1* knockout mice at 28 days post injury, a finding similar to that in the HSALR mice (Figure S6). This suggests that functional deficits in MBNL1, either by sequestration of MBNL1 by RNA foci (HSALR mice), or the loss of *Mbnl1* expression (Mbnl1 knockout), can lead to the multiple central nuclei phenotype in regenerated muscle.

**Figure 9:**
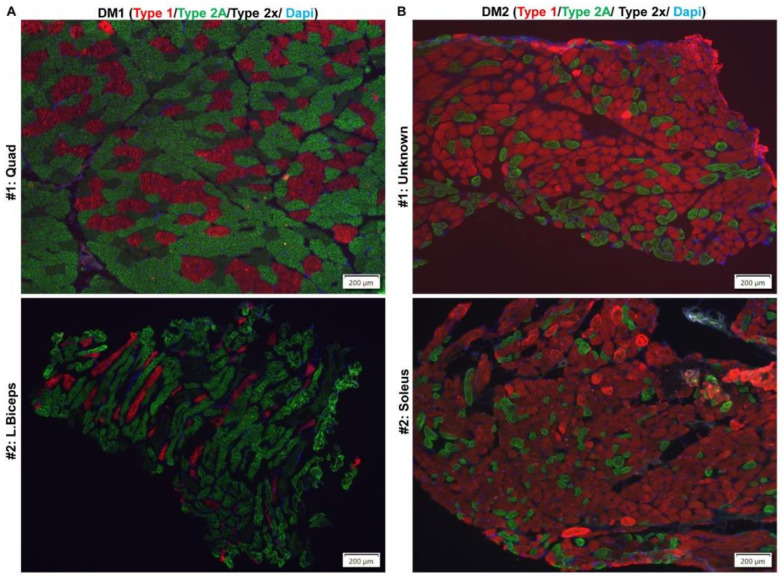
Representative images of immunofluorescence staining for MHC type 1 (red), type 2A (green), and type IIX (no staining) from (**A**) DM1 and (**B**) DM2 skeletal muscles. The Type 1 (MYH7) fibers are stained with antibody BA-D5 (DSHB), and the Type 2A (MYH2) fibers are stained with antibody SC-71 (DSHB). Nuclei are stained with DAPI (blue). Scale bars are shown.

**Figure 10:**
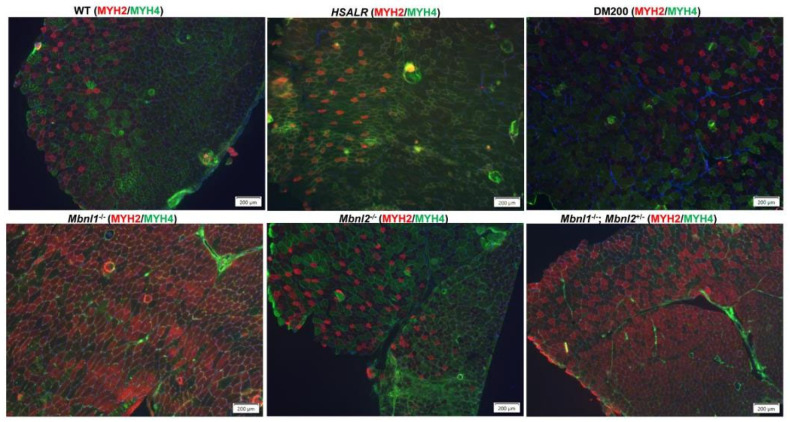
Immunofluorescence staining of muscle fiber types using an anti-MYH2 antibody (red) for type IIA and an anti-MYH4 antibody for type IIB (green) in the tibialis anterior (TA) muscle sections of different mouse models under non-damaged conditions (saline). Nuclei are stained with DAPI (blue). Scale bars = 200 μm.

**Figure 11:**
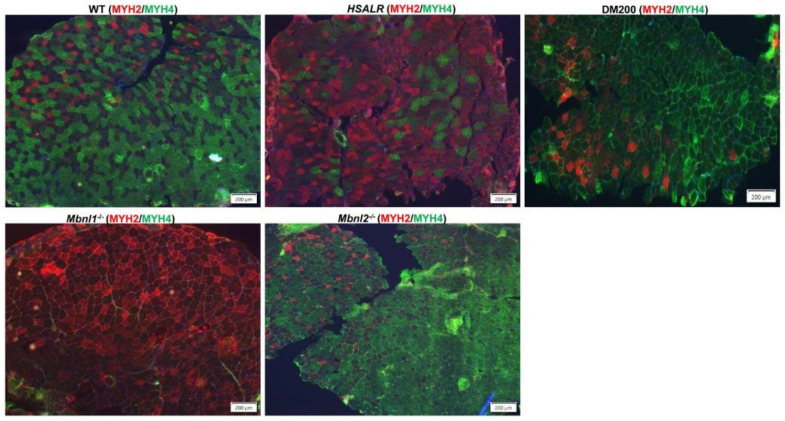
Immunofluorescence staining of muscle fiber types using an anti-MYH2 antibody (red) for type IIA and an anti-MYH4 antibody for type IIB (green) in tibialis anterior (TA) muscle sections 28 days after barium chloride (BaCl_2_) injury of different mouse models. Nuclei are stained with DAPI (blue). Scale bars = 200 μm.

Another significant finding of this study is the switch in fiber type proportions after damage. The muscles in the human body are composed of a mixture of fiber types that exhibit both contractile (slow-twitch or fast-twitch) and metabolic (oxidative or glycolytic) properties. The muscle fiber type distribution can be disrupted with age and disease. In DM1, typically there is type 1 fiber atrophy (MYH7) and type 2 fiber hypertrophy [58,59,60] (Pakleza-Foila Morphol 2011;Vihola-2003), whereas DM2 shows preferential atrophy of type 2 fibers resulting in a relative abundance of type 1 fibers (Figure 9) [58,59,61]. It is challenging to study the muscle fiber switch in mice due to the relative lack of type 1 fibers in most murine muscles including the TA muscle. In the TA muscle of mice, type 2 fibers (Myh2 (IIA), Myh4 (IIB), and Myh1 (IIX) are expressed. Type IIA (Myh2) fibers are more oxidative than Type IIB (Myh4) fibers. In our study, the fiber type proportion in undamaged TA muscles from HSALR mice was similar to that in WT mice (Figure 4). The fraction of oxidative fibers (type IIA, Myh2) is increased in regenerated TA muscles from HSALR mice, whereas the fraction of glycolytic fibers (type IIB, Myh4) is decreased. This was not seen with WT mice. We reevaluated fiber type distribution in various models and found that undamaged TA from HSALR, DM200, and *Mbnl2^−/−^* mice are similar to WT TA muscles with a predominance of Type IIB fibers (Figure 10). However, TA muscles from *Mbnl1^−/−^* and *Mbnl1^−/−^/Mbnl2^+/−^* mice showed a dramatic fiber type switch to Type IIA predominance. After induced damage, only HSALR TA muscles showed a noticeable fiber type switch, such that they now resembled TA muscles from *Mbnl1^−/−^* mice (Figure 11). These results demonstrate a substantial fiber type switch to more oxidative fibers. This resembles the human DM2 condition, characterized by a reduced fraction of glycolytic fibers, and a marked shift to more oxidative fiber types (see Figure 9 and Figure 11) [59]. This contrasts with the changes in DM1 and the DM200 model, which are more similar to each other. This may be a reflection of what cell types and fiber types the toxic RNA is expressed in as well as its nature (i.e., CUG repeats in the context of *HSA* 3′UTR versus the *DMPK* 3′UTR).

In conclusion, this study shows that RNA toxicity in HSALR mice results in an increased proportion of larger muscle fibers and a concomitant decrease in fiber number in the TA muscle and correlates with decreased grip strength with time. This is accompanied by a slightly increased percentage of centronucleated fibers (Figure 1). We also found increased numbers of MuSCs and increased expression of *Pax7*. However, this increase in MuSCs did not seem to be related to RNA toxicity, as knockdown of the toxic RNA levels in HSALR mice had no effect on this (Figure S3). Overall, muscle regeneration in HSALR mice restores the muscle histology back to its baseline (Figure 2). Early steps in muscle regeneration are intact in HSALR mice (Figure 3). But, at the end of the regenerative process, HSALR TA muscles had more fibers with more central nuclei and exhibited a fiber type switch towards more oxidative fibers (Figure 4). There was no evidence of increased fibrosis after one round of regeneration (Figure 4). The duration of RNA toxicity (2 vs. 6 months) did not seem to make a difference in the regenerative response (Figure 5, Figure 6 and Figure 7). The cause of the increased centronucleation is unclear. Evaluation of two candidate splicing events of genes known to play a role in nuclear positioning (*Bin1* and *Syne 1)* provided no correlation (Figures S4 and S5). A logical thought is that the increased centronucleation after regeneration might be more directly related to the increased numbers of MuSCs and activated MuSCs in HSALR mice, contributing to increased nuclear accretion. Lastly, one of the more remarkable findings is the fiber type switching that occurs in the regenerated TA muscles of HSALR mice. Interestingly, this resembles more closely the fiber type switching in DM2 than in DM1. Importantly, *Mbnl1^−/−^* mice also exhibit this fiber type switch and increased centronucleation (Figure 10, Figure 11 and Figure S6). So, it may be that during the regenerative process, increased toxic RNA expression in the maturing fiber (remembering it is being driven by the HSA promoter) is not matched by a concomitant increase in MBNL1, resulting in a more effective functional deficit of MBNL1 than in the undamaged adult HSALR muscle. This gives scope for future investigations!

## 4. Materials and Methods

### 4.1. Experimental Mice

We used all animals under protocols approved by the University of Virginia’s Animal Care and Use Committee. This study used WT mice (FVB) and an HSALR line (LR20b) (Strain#:032031, Jackson Laboratory, Bar Harbor, ME, USA). Wildtype (FVB) (Strain#:001800) mice, 2 to 6 months old, purchased from Jackson Laboratory, were used as a control for HSALR mice. We used both males and females for all the studies.

### 4.2. Forelimb Grip Strength Test

We used a digital grip strength meter (GSM) (0167–005L, Columbus Instruments, Columbus, OH, USA) to measure forelimb grip strength. The GSM records an animal’s maximal strength in peak tension mode (T-PK) while resisting an opposing pulling force. In the measuring grip strength test, a mouse was allowed to grasp the bar mounted on the force gauge. The digital force transducer records the peak pull force in grams force (gf), the average of five consecutive grams of force on the same day for each animal was used for analysis. We also present data as a weight-normalized forelimb grip strength, grams force/gram (gf/g).

### 4.3. Skeletal Muscle Injury

For induced muscle damage, at the age of 2 and 6 months, 50 μL of 1.2% BaCl_2_ (Sigma-Aldrich, St. Louis, MO, USA) dissolved in saline was injected into the right tibialis anterior (TA) muscle of mice at three different places (proximal/mid/distal) to induce necrotic injury. We injected saline into the left TA muscle in the opposite leg of each mouse as a non-injury control. Mice were euthanized, and TA muscles were collected at 0, 5, 7, 10, 14, and 28 days post injury (dpi) for immunofluorescence, RNA analysis, and histological analyses, as indicated in the results section.

### 4.4. H&E Staining and Muscle Fiber Analysis of Skeletal Muscles

We froze muscles (tibialis anterior, TA) in liquid nitrogen-cooled isopentene. Ten-micrometer muscle sections were stained with H&E according to standard protocols and examined under a light microscope. We conducted microscopy using an Olympus IX 50 inverted microscope and captured the images using a CCD camera. At least three non-overlapping sections per mouse were analyzed, and 4–6 mice per group were analyzed.

We performed muscle fiber size analysis using Cellpose combined with LabelsToRois by measuring the cross-sectional area of each muscle fiber in digital images of H&E-stained skeletal muscle (tibialis anterior) sections captured at 100× magnification. Fibers were also counted using the same software [62,63]. A representative H&E-stained image of the tibialis anterior (TA) muscle showing fibers with different numbers of internal nuclei used for counting purposes is presented in Figure S7. Fibers with three or more internal nuclei were counted together. At least 3–5 non-overlapping sections per mouse were analyzed, and 3–5 mice per group were analyzed.

### 4.5. RNA Extraction, Quantitative Real-Time PCR (qRT-PCR) and Splicing Analysis

Using published protocols, we extracted total RNA from skeletal muscle (tibialis anterior, TA) tissues [64]. All RNAs are DNAse-treated and confirmed to be free of DNA contamination. cDNA was made from 1 µg of total RNA using QuantiTech^TM^ Reverse Transcription Kit (Qiagen^TM^, Germantown, MD, USA). We performed quantitative RT-PCR to determine gene expression using the BioRad iCycler^TM^ (Biorad, Hercules, CA, USA) and detected with SYBER^TM^ Green dye. Data were normalized using an endogenous control (*Gapdh*), and normalized values were subjected to a 2^−ΔΔCt^ formula to calculate the fold changes between groups. Results having a Ct value over 35 were considered as being below detection limits. Tables S1 and S2 provides the primer sequences and conditions used in this study.

### 4.6. RNA-FISH and Immunofluorescence

For RNA-FISH, we fixed tissue in 4% paraformaldehyde in 1× PBS and used a Cy3-CAG10 (Integrated DNA technology, IDT) probe for hybridization. Details about the RNA-FISH protocols are described elsewhere [65]. Immunofluorescence was performed as described previously [65]. Primary antibodies were anti-PAX7 (1:20, PAX7-c, DSHB), anti-MYH3 (1:200, clone F1.652, DSHB), anti-MYH2(1:20, SC-71-c, DSHB), anti-MYH4 (1:10, BF-F3-c, DSHB), anti-MYH7 (1:20, BA-D5 (DSHB), anti-laminin (1:1000, catalog L9393, Sigma-Aldrich), and anti-MyoD rabbit polyclonal (1:200, NBP1-54153, Novus, Chesterfield, MO, USA). Secondary antibodies were from Molecular Probes (1:500 dilution) (Eugene, OR, USA). For all quantification of PAX7+ cells by IF, we used co-staining with laminin and DAPI on at least three non-overlapping sections per mouse and 3–5 mice per group.

### 4.7. Statistical Analysis

We used standard statistics to analyze the data. Briefly, data sets were first analyzed for outliers using Grubb’s test. For real-time PCR analysis, we first assessed outliers before the fold change calculation. After removing outliers, we analyzed the data set for normality. If normal, the data were analyzed using an unpaired Student’s *t*-test with equal or unequal variance as appropriate. All data are expressed as mean ± SEM. * *p* < 0.05, ** *p* < 0.01, and *** *p* <0.001 (Student’s *t*-test). Unless otherwise specified, we considered values less than *p* < 0.05 to be statistically significant. ns means not significant.

## Data Availability

The original contributions presented in this study are included in the article/Supplementary Materials. Further inquiries can be directed to the corresponding authors.

## Supplementary Materials

The following supporting information can be downloaded at: https://www.mdpi.com/article/10.3390/ijms262210985/s1.

## References

[1] Johnson N.E., Butterfield R.J., Mayne K., Newcomb T., Imburgia C., Dunn D., Duval B., Feldkamp M.L., Weiss R.B. (2021). Population-Based Prevalence of Myotonic Dystrophy Type 1 Using Genetic Analysis of Statewide Blood Screening Program. Neurology.

[2] Liao Q., Zhang Y., He J., Huang K. (2022). Global Prevalence of Myotonic Dystrophy: An Updated Systematic Review and Meta-Analysis. Neuroepidemiology.

[3] Mahadevan M., Tsilfidis C., Sabourin L., Shutler G., Amemiya C., Jansen G., Neville C., Narang M., Barcelo J., O’Hoy K. (1992). Myotonic dystrophy mutation: An unstable CTG repeat in the 3′ untranslated region of the gene. Science.

[4] Brook J.D., McCurrach M.E., Harley H.G., Buckler A.J., Church D., Aburatani H., Hunter K., Stanton V.P., Thirion J.P., Hudson T. (1992). Molecular basis of myotonic dystrophy: Expansion of a trinucleotide (CTG) repeat at the 3′ end of a transcript encoding a protein kinase family member. Cell.

[5] Taneja K.L., McCurrach M., Schalling M., Housman D., Singer R.H. (1995). Foci of trinucleotide repeat transcripts in nuclei of myotonic dystrophy cells and tissues. J. Cell Biol..

[6] Davis B.M., McCurrach M.E., Taneja K.L., Singer R.H., Housman D.E. (1997). Expansion of a CUG trinucleotide repeat in the 3′ untranslated region of myotonic dystrophy protein kinase transcripts results in nuclear retention of transcripts. Proc. Natl. Acad. Sci. USA.

[7] Mankodi A., Takahashi M.P., Jiang H., Beck C.L., Bowers W.J., Moxley R.T., Cannon S.C., Thornton C.A. (2002). Expanded CUG repeats trigger aberrant splicing of ClC-1 chloride channel pre-mRNA and hyperexcitability of skeletal muscle in myotonic dystrophy. Mol. Cell.

[8] Osborne R.J., Thornton C.A. (2006). RNA-dominant diseases. Hum. Mol. Genet..

[9] Jones K., Wei C., Iakova P., Bugiardini E., Schneider-Gold C., Meola G., Woodgett J., Killian J., Timchenko N.A., Timchenko L.T. (2012). GSK3β mediates muscle pathology in myotonic dystrophy. J. Clin. Investig..

[10] Kuyumcu-Martinez N.M., Wang G.S., Cooper T.A. (2007). Increased steady-state levels of CUGBP1 in myotonic dystrophy 1 are due to PKC-mediated hyperphosphorylation. Mol. Cell.

[11] Ravel-Chapuis A., Belanger G., Yadava R.S., Mahadevan M.S., DesGroseillers L., Cote J., Jasmin B.J. (2012). The RNA-binding protein Staufen1 is increased in DM1 skeletal muscle and promotes alternative pre-mRNA splicing. J. Cell Biol..

[12] Brockhoff M., Rion N., Chojnowska K., Wiktorowicz T., Eickhorst C., Erne B., Frank S., Angelini C., Furling D., Ruegg M.A. (2017). Targeting deregulated AMPK/mTORC1 pathways improves muscle function in myotonic dystrophy type I. J. Clin. Investig..

[13] Yadava R.S., Foff E.P., Yu Q., Gladman J.T., Zheng T.S., Mahadevan M.S. (2016). TWEAK Regulates Muscle Functions in a Mouse Model of RNA Toxicity. PLoS ONE.

[14] Yadava R.S., Foff E.P., Yu Q., Gladman J.T., Kim Y.K., Bhatt K.S., Thornton C.A., Zheng T.S., Mahadevan M.S. (2015). TWEAK/Fn14, a pathway and novel therapeutic target in myotonic dystrophy. Hum. Mol. Genet..

[15] Kim D.H., Langlois M.A., Lee K.B., Riggs A.D., Puymirat J., Rossi J.J. (2005). HnRNP H inhibits nuclear export of mRNA containing expanded CUG repeats and a distal branch point sequence. Nucleic Acids Res..

[16] Pettersson O.J., Aagaard L., Andrejeva D., Thomsen R., Jensen T.G., Damgaard C.K. (2014). DDX6 regulates sequestered nuclear CUG-expanded DMPK-mRNA in dystrophia myotonica type 1. Nucleic Acids Res..

[17] Laurent F.X., Sureau A., Klein A.F., Trouslard F., Gasnier E., Furling D., Marie J. (2012). New function for the RNA helicase p68/DDX5 as a modifier of MBNL1 activity on expanded CUG repeats. Nucleic Acids Res..

[18] Llamusi B., Bargiela A., Fernandez-Costa J.M., Garcia-Lopez A., Klima R., Feiguin F., Artero R. (2013). Muscleblind, BSF and TBPH are mislocalized in the muscle sarcomere of a Drosophila myotonic dystrophy model. Dis. Model. Mech..

[19] Ozimski L.L., Sabater-Arcis M., Bargiela A., Artero R. (2021). The hallmarks of myotonic dystrophy type 1 muscle dysfunction. Biol. Rev. Camb. Philos. Soc..

[20] Mankodi A., Logigian E., Callahan L., McClain C., White R., Henderson D., Krym M., Thornton C.A. (2000). Myotonic dystrophy in transgenic mice expressing an expanded CUG repeat. Science.

[21] Orengo J.P., Chambon P., Metzger D., Mosier D.R., Snipes G.J., Cooper T.A. (2008). Expanded CTG repeats within the DMPK 3′ UTR causes severe skeletal muscle wasting in an inducible mouse model for myotonic dystrophy. Proc. Natl. Acad. Sci. USA.

[22] Mahadevan M.S., Yadava R.S., Yu Q., Balijepalli S., Frenzel-McCardell C.D., Bourne T.D., Phillips L.H. (2006). Reversible model of RNA toxicity and cardiac conduction defects in myotonic dystrophy. Nat. Genet..

[23] Ho T.H., Bundman D., Armstrong D.L., Cooper T.A. (2005). Transgenic mice expressing CUG-BP1 reproduce splicing mis-regulation observed in myotonic dystrophy. Hum. Mol. Genet..

[24] Kanadia R.N., Johnstone K.A., Mankodi A., Lungu C., Thornton C.A., Esson D., Timmers A.M., Hauswirth W.W., Swanson M.S. (2003). A muscleblind knockout model for myotonic dystrophy. Science.

[25] Timchenko N.A., Patel R., Iakova P., Cai Z.J., Quan L., Timchenko L.T. (2004). Overexpression of CUG triplet repeat-binding protein, CUGBP1, in mice inhibits myogenesis. J. Biol. Chem..

[26] Ward A.J., Rimer M., Killian J.M., Dowling J.J., Cooper T.A. (2010). CUGBP1 overexpression in mouse skeletal muscle reproduces features of myotonic dystrophy type 1. Hum. Mol. Genet..

[27] Seznec H., Agbulut O., Sergeant N., Savouret C., Ghestem A., Tabti N., Willer J.C., Ourth L., Duros C., Brisson E. (2001). Mice transgenic for the human myotonic dystrophy region with expanded CTG repeats display muscular and brain abnormalities. Hum. Mol. Genet..

[28] Ciciliot S., Rossi A.C., Dyar K.A., Blaauw B., Schiaffino S. (2013). Muscle type and fiber type specificity in muscle wasting. Int. J. Biochem. Cell Biol..

[29] Casar J.C., McKechnie B.A., Fallon J.R., Young M.F., Brandan E. (2004). Transient up-regulation of biglycan during skeletal muscle regeneration: Delayed fiber growth along with decorin increase in biglycan-deficient mice. Dev. Biol..

[30] Hardy D., Besnard A., Latil M., Jouvion G., Briand D., Thepenier C., Pascal Q., Guguin A., Gayraud-Morel B., Cavaillon J.M. (2016). Comparative Study of Injury Models for Studying Muscle Regeneration in Mice. PLoS ONE.

[31] Caldwell C.J., Mattey D.L., Weller R.O. (1990). Role of the basement membrane in the regeneration of skeletal muscle. Neuropathol. Appl. Neurobiol..

[32] Yadava R.S., Mandal M., Giese J.M., Rigo F., Bennett C.F., Mahadevan M.S. (2021). Modeling muscle regeneration in RNA toxicity mice. Hum. Mol. Genet..

[33] Yadava R.S., Mandal M., Mahadevan M.S. (2024). Studying the Effect of MBNL1 and MBNL2 Loss in Skeletal Muscle Regeneration. Int. J. Mol. Sci..

[34] Andersen G., Orngreen M.C., Preisler N., Colding-Jorgensen E., Clausen T., Duno M., Jeppesen T.D., Vissing J. (2013). Muscle phenotype in patients with myotonic dystrophy type 1. Muscle Nerve.

[35] Bassez G., Chapoy E., Bastuji-Garin S., Radvanyi-Hoffman H., Authier F.J., Pellissier J.F., Eymard B., Gherardi R.K. (2008). Type 2 myotonic dystrophy can be predicted by the combination of type 2 muscle fiber central nucleation and scattered atrophy. J. Neuropathol. Exp. Neurol..

[36] Augusto V., Padovani C., Eduardo G., Campos R. (2004). Skeletal muscle fiber types in C57BL6J mice. J. Morphol. Sci..

[37] Mauro A. (1961). Satellite cell of skeletal muscle fibers. J. Biophys. Biochem. Cytol..

[38] von Maltzahn J., Jones A.E., Parks R.J., Rudnicki M.A. (2013). Pax7 is critical for the normal function of satellite cells in adult skeletal muscle. Proc. Natl. Acad. Sci. USA.

[39] Sahgal V., Bernes S., Sahgal S., Lischwey C., Subramani V. (1983). Skeletal muscle in preterm infants with congenital myotonic dystrophy. Morphologic and histochemical study. J. Neurol. Sci..

[40] Thornell L.E., Lindstom M., Renault V., Klein A., Mouly V., Ansved T., Butler-Browne G., Furling D. (2009). Satellite cell dysfunction contributes to the progressive muscle atrophy in myotonic dystrophy type 1. Neuropathol. Appl. Neurobiol..

[41] Yadava R.S., Kim Y.K., Mandal M., Mahadevan K., Gladman J.T., Yu Q., Mahadevan M.S. (2019). MBNL1 overexpression is not sufficient to rescue the phenotypes in a mouse model of RNA toxicity. Hum. Mol. Genet..

[42] Pizza F.X., Buckley K.H. (2023). Regenerating Myofibers after an Acute Muscle Injury: What Do We Really Know about Them?. Int. J. Mol. Sci..

[43] Fukada S.I., Higashimoto T., Kaneshige A. (2022). Differences in muscle satellite cell dynamics during muscle hypertrophy and regeneration. Skelet. Muscle.

[44] Cutler A.A., Pawlikowski B., Wheeler J.R., Betta N.D., Elston T., O’Rourke R., Jones K., Olwin B.B. (2022). The regenerating skeletal muscle niche drives satellite cell return to quiescence. iScience.

[45] Buckley K.H., Nestor-Kalinoski A.L., Pizza F.X. (2022). Positional Context of Myonuclear Transcription During Injury-Induced Muscle Regeneration. Front. Physiol..

[46] Hojfeldt G., Sorenson T., Gonzales A., Kjaer M., Andersen J.L., Mackey A.L. (2023). Fusion of myofibre branches is a physiological feature of healthy human skeletal muscle regeneration. Skelet. Muscle..

[47] Wada K., Katsuta S., Soya H. (2008). Formation process and fate of the nuclear chain after injury in regenerated myofiber. Anat. Rec..

[48] Meyer G.A. (2018). Evidence of induced muscle regeneration persists for years in the mouse. Muscle Nerve.

[49] Gómez-Oca R., Cowling B.S., Laporte J. (2021). Common Pathogenic Mechanisms in Centronuclear and Myotubular Myopathies and Latest Treatment Advances. Int. J. Mol. Sci..

[50] Fugier C., Klein A.F., Hammer C., Vassilopoulos S., Ivarsson Y., Toussaint A., Tosch V., Vignaud A., Ferry A., Messaddeq N. (2011). Misregulated alternative splicing of BIN1 is associated with T tubule alterations and muscle weakness in myotonic dystrophy. Nat. Med..

[51] Kimura T., Nakamori M., Lueck J.D., Pouliquin P., Aoike F., Fujimura H., Dirksen R.T., Takahashi M.P., Dulhunty A.F., Sakoda S. (2005). Altered mRNA splicing of the skeletal muscle ryanodine receptor and sarcoplasmic/endoplasmic reticulum Ca^2+^-ATPase in myotonic dystrophy type 1. Hum. Mol. Genet..

[52] Böhm J., Vasli N., Maurer M., Cowling B.S., Shelton G.D., Kress W., Toussaint A., Prokic I., Schara U., Anderson T.J. (2013). Altered splicing of the BIN1 muscle-specific exon in humans and dogs with highly progressive centronuclear myopathy. PLoS Genet..

[53] Prokic I., Cowling B.S., Kutchukian C., Kretz C., Tasfaout H., Gache V., Hergueux J., Wendling O., Ferry A., Toussaint A. (2020). Differential physiological roles for BIN1 isoforms in skeletal muscle development, function and regeneration. Dis. Model. Mech..

[54] Hintze S., Knaier L., Limmer S., Schoser B., Meinke P. (2018). Nuclear Envelope Transmembrane Proteins in Myotonic Dystrophy Type 1. Front. Physiol..

[55] Todorow V., Hintze S., Schoser B., Meinke P. (2022). Nuclear envelope transmembrane proteins involved in genome organization are misregulated in myotonic dystrophy type 1 muscle. Front. Cell Dev. Biol..

[56] Apel E.D., Lewis R.M., Grady R.M., Sanes J.R. (2000). Syne-1, a dystrophin- and Klarsicht-related protein associated with synaptic nuclei at the neuromuscular junction. J. Biol. Chem..

[57] Zhang X., Xu R., Zhu B., Yang X., Ding X., Duan S., Xu T., Zhuang Y., Han M. (2007). Syne-1 and Syne-2 play crucial roles in myonuclear anchorage and motor neuron innervation. Development.

[58] Nadaj-Pakleza A., Lusakowska A., Sułek-Piątkowska A., Krysa W., Rajkiewicz M., Kwieciński H., Kamińska A. (2011). Muscle pathology in myotonic dystrophy: Light and electron microscopic investigation in eighteen patients. Folia Morphol..

[59] Schoser B.G., Schneider-Gold C., Kress W., Goebel H.H., Reilich P., Koch M.C., Pongratz D.E., Toyka K.V., Lochmuller H., Ricker K. (2004). Muscle pathology in 57 patients with myotonic dystrophy type 2. Muscle Nerve.

[60] Vihola A., Bassez G., Meola G., Zhang S., Haapasalo H., Paetau A., Mancinelli E., Rouche A., Hogrel J.Y., Laforet, P. (2003). Histopathological differences of myotonic dystrophy type 1 (DM1) and PROMM/DM2. Neurology.

[61] Andre L.M., Ausems C.R., Wansink D.G., Wieringa B. (2018). Abnormalities in Skeletal Muscle Myogenesis, Growth, and Regeneration in Myotonic Dystrophy. Front. Neurol..

[62] Stringer C., Wang T., Michaelos M., Pachitariu M. (2021). Cellpose: A generalist algorithm for cellular segmentation. Nat. Methods.

[63] Waisman A., Norris A.M., Elias Costa M., Kopinke D. (2021). Automatic and unbiased segmentation and quantification of myofibers in skeletal muscle. Sci. Rep..

[64] Langlois M.A., Lee N.S., Rossi J.J., Puymirat J. (2003). Hammerhead ribozyme-mediated destruction of nuclear foci in myotonic dystrophy myoblasts. Mol. Ther..

[65] Mankodi A., Teng-Umnuay P., Krym M., Henderson D., Swanson M., Thornton C.A. (2003). Ribonuclear inclusions in skeletal muscle in myotonic dystrophy types 1 and 2. Ann. Neurol..

