# Supplementary material for: Multiple Defects in Muscle Regeneration in the HSALR Mouse Model of RNA Toxicity
Source: Int J Mol Sci. 2025 Nov 13;26(22):10985. doi: 10.3390/ijms262210985 (PMC12651964; doi:10.3390/ijms262210985)
Supplement: Supplementary file 1 [file ijms-26-10985-s001.zip › ijms-3865883-supplementary.pdf]

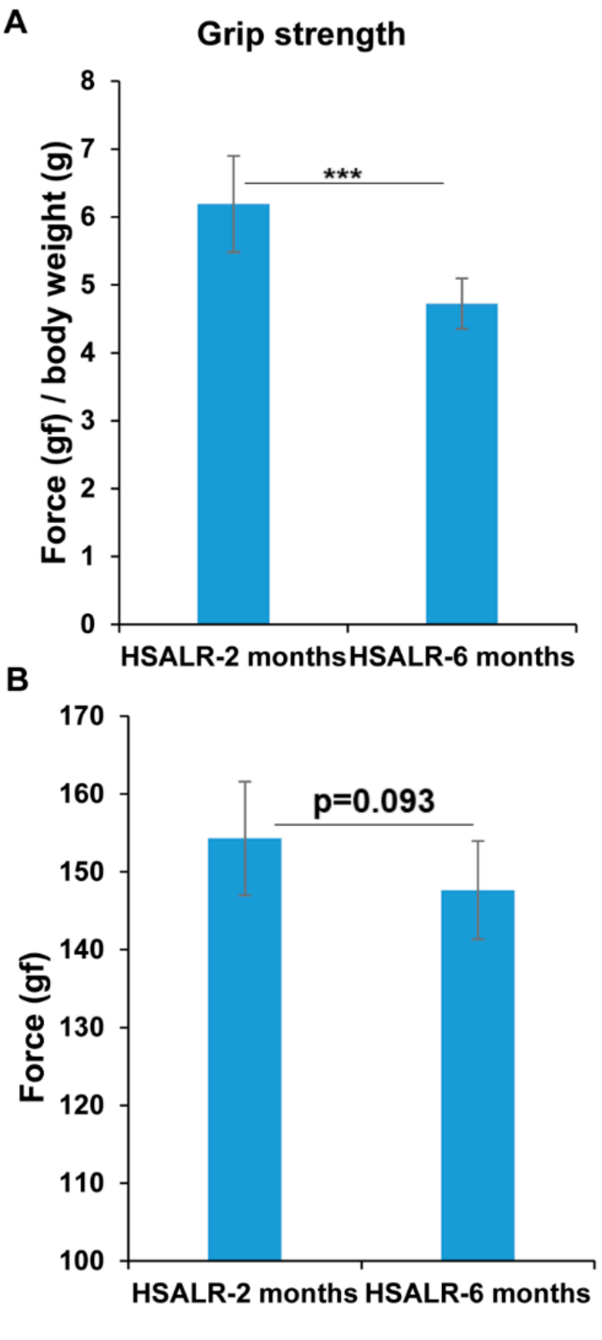

Figure S1: Grip strength analysis of *HSA*^{LR} mice at 2 months and 6 months of age. The number of mice used at 2 months of age was seven (four females and three males). The same mice were followed till they were 6 months of age. **A**) The peak pull force measured in grams force adjusted with the body weight of mice, grams force/gram (gf/g). ***$p<0.001$. **B**) The graph represents the peak pull force (gf) without weight adjustment. Error bars are mean±STDEV; p values are indicated.

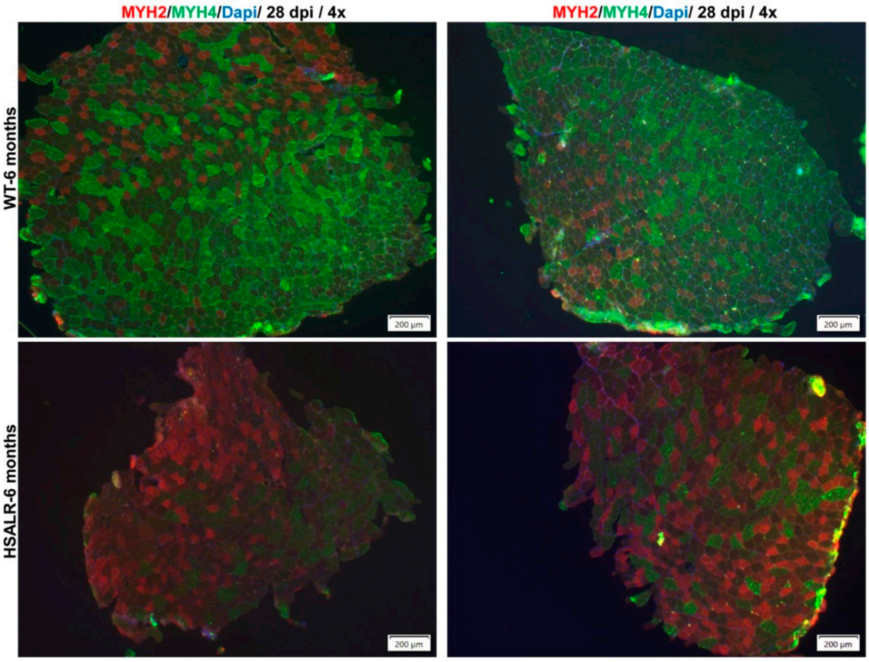

Figure S2: These are lower-magnification pictures of the data presented in Figure 4. MYH2 IF (Red) and MYH4 IF (green) in TA muscle sections 28 days post-damage ($BaCl_2$) show an increased number of MYH2 (oxidative) fibers in *HSA*^{LR} mice compared to wild-type mice. Nuclei are stained with DAPI (blue). Scale bars are shown.

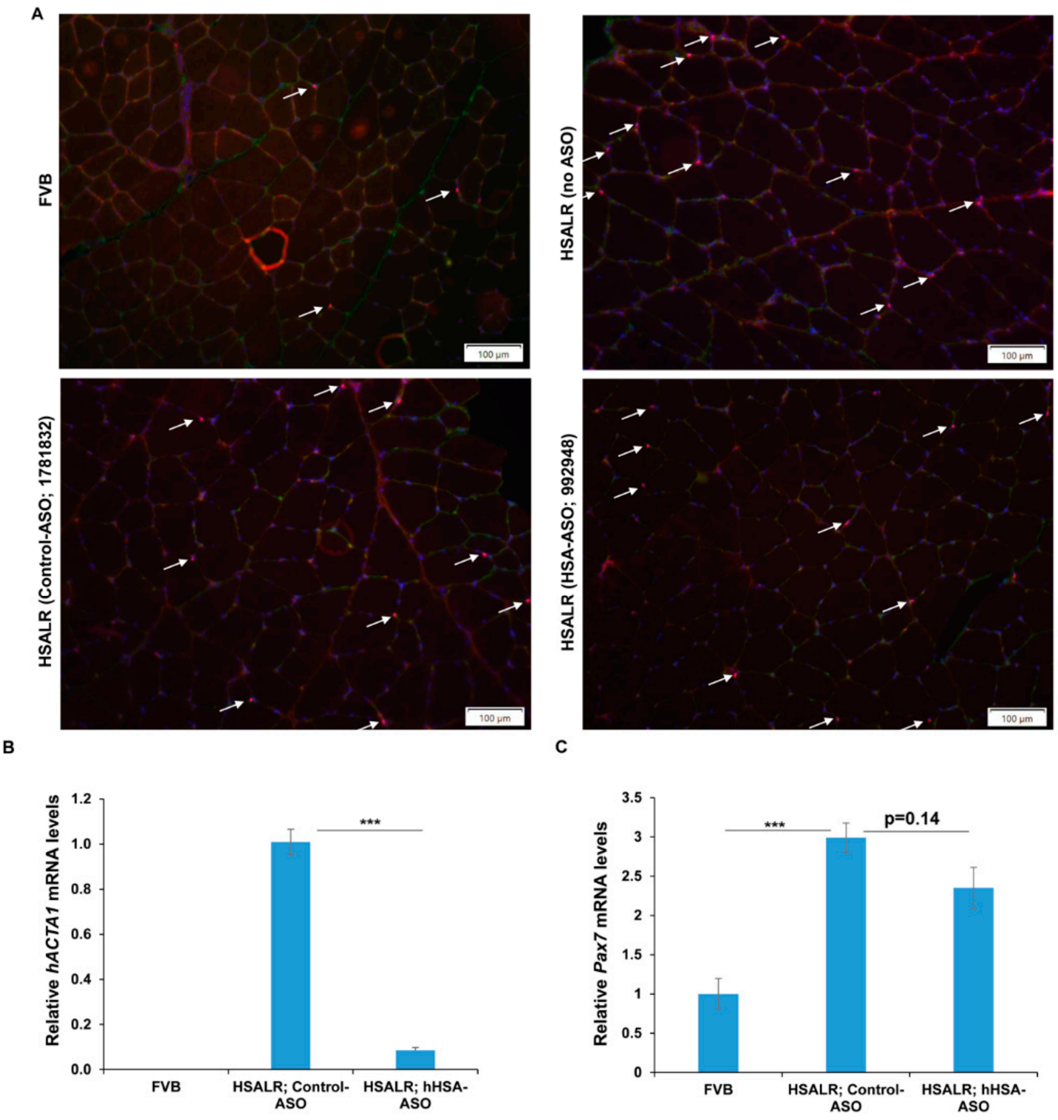

Figure S3: The increased number of MuSCs in *HSA*^{LR} mice is not affected by antisense oligonucleotide treatment. **A)** PAX7-IF was used to detect MuSCs (white arrows) in the quadriceps femoris of mice in the indicated group. DAPI was used to stain nuclei (blue). **B**) qRT-PCR shows reduced *hACAT1* mRNA in skeletal muscle (quadriceps femoris) of *HSA*^{LR} mice treated with hHSA-ASO (#992948) as compared to control ASO-treated mice. **C**) qRT-PCR shows no significant reduction of *Pax7* mRNA in the quadriceps femoris muscle treated with hHSA-ASO (#992948) as compared to control ASO-treated mice. N=5-7 mice /group, ***p<0.001; Student's t-test; Error bars are mean±SEM.

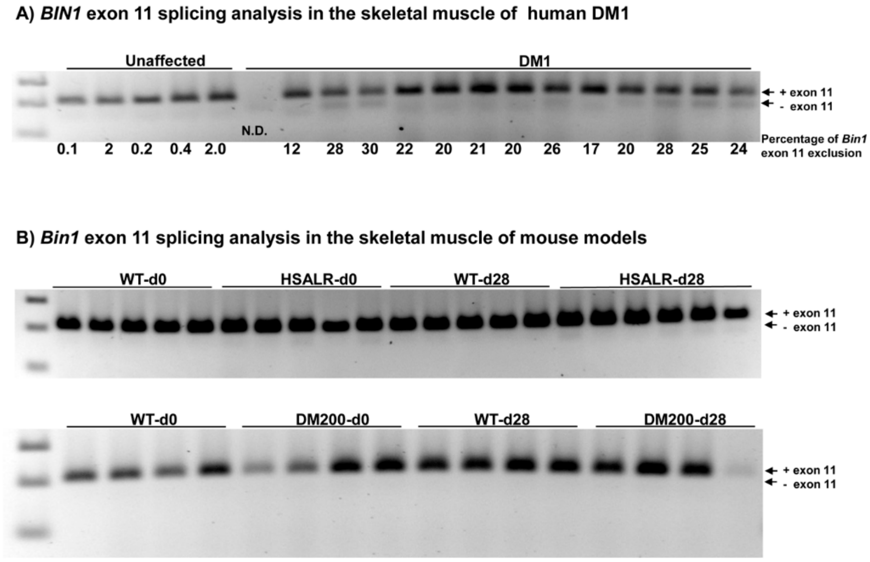

Figure S4: Analysis of *BIN1*/*Bin1* exon 11 RNA splicing. **(**A**)** RT-PCR analysis of *BIN1* exon 11 splicing in the skeletal muscle of human DM1. The quantification of *BIN1* mRNA exon 11-ve isoform in skeletal muscle samples from individuals with DM1 is shown below the picture. N.D. means not detected. (B) RT-PCR analysis of *Bin1* exon 11 splicing in the skeletal muscle of mouse models (HSALR and DM200) with damaged (d28: 28 dpi) and non-damaged (d0) conditions. We could not quantitatively determine *Bin1* mRNA exon -11ve isoform in the samples from the mouse models because of the low detectable levels of products. The details about primers and conditions are given in Table S2.

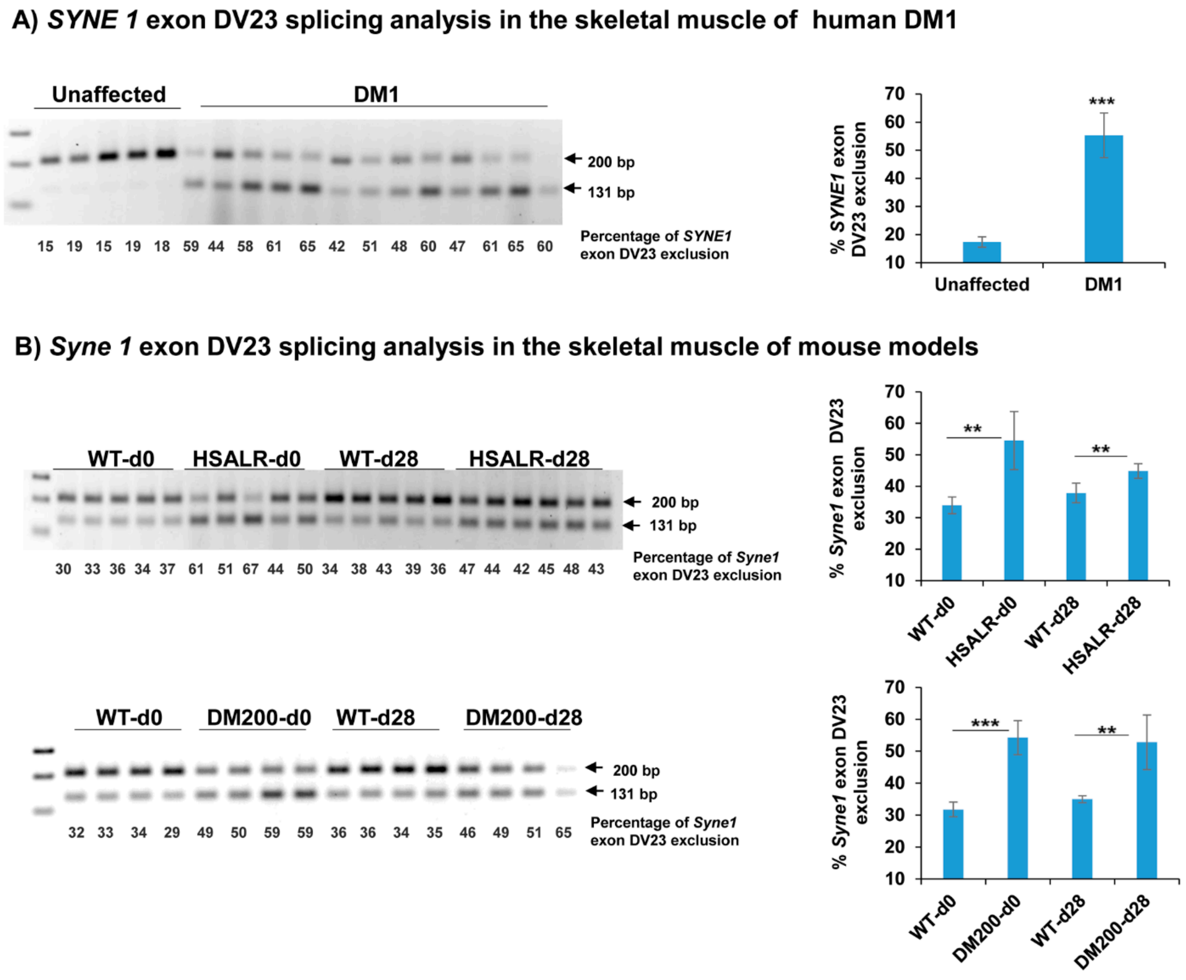

Figure S5: Analysis of RNA splicing. RT-PCR analysis of (**A**) *SYNE1* muscle-specific exon DV23 in the skeletal muscle of human DM1 and (**B**) *Syne1* muscle-specific exon DV23 in the skeletal muscle of mouse models (HSALR and DM200) with damaged (d28: 28 dpi) and non-damaged (d0) conditions. The bands reflect the alternative spliced forms that differ by 69 base pairs, which is also called the DV23 exon. The quantification of exon DV23 -ve isoform is also shown below for each gel. Primers and conditions are given in Table S2.

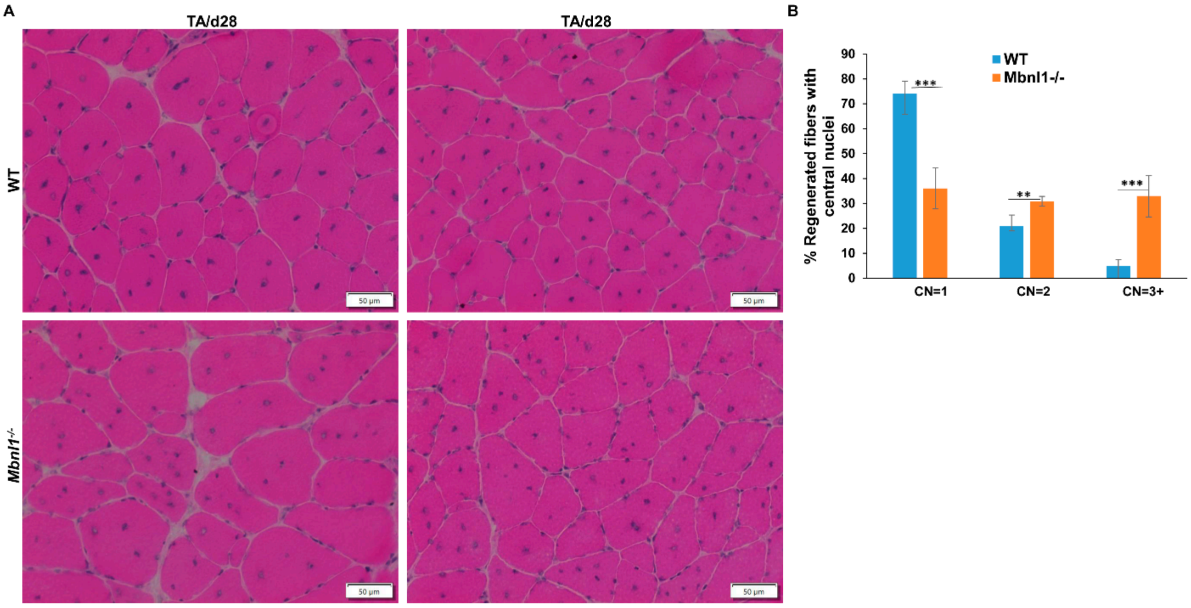

Figure S6: TA (tibialis anterior) muscle in WT and *Mbnl1*$^{-/-}$ mice at 28 days post-injury. **(A)** Representative image of H&E-stained TA muscle sections 28 days post-injury (dpi). Scale bars = 50 µm. **(B)** The percentage of regenerated fibers with central nuclei (CN=1, p=0.000227) (CN=2, p=0.006067), (CN=>3, p=0.000669) (CN=>2, p=0.000227), WT=1618 fibers, *Mbnl1*$^{-/-}$=1351 fibers. CN: central nuclei per fiber; n= 4 mice/group; **p<0.01, ***p<0.001; Student's t-test; error bars are mean±SEM.

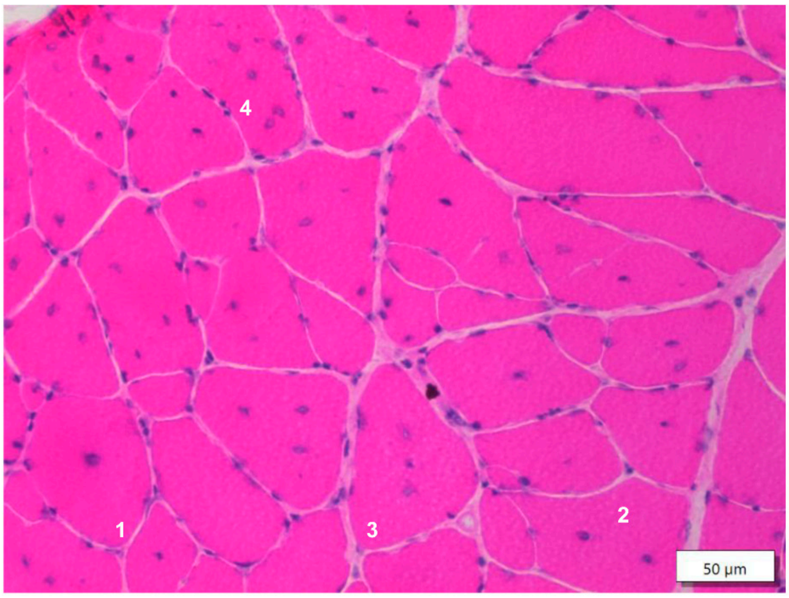

1= fiber with one central nuclei (CN=1)
2= fiber with two central nuclei (CN=2)
3= fiber with three central nuclei (CN=3)
4= fiber with more than three central nuclei (CN=>3)

Figure S7: A representative image of H&E-stained TA muscle showing fibers #1: one internal nucleus (CN=1), fibers#2: two internal nuclei (CN=2), fibers# 3: three internal

nuclei (CN=3), and fibers#4: more than three nuclei. Fibers with three or more internal nuclei were counted together (CN=>3).

Table S1. Primers for real-time RT-PCR

Gene	Forward primer	Reverse primer	Anneal-ing temp (°C)	PCR effi-ciency (%)
Gapdh	AGGTCGGTGTGAACGGATTTG	TGTAGACCATGTAGTTGAGGT CA	62	93
Pax7	AGGACGACGAGGAAGGAGACA	TCATCCAGACGGTTCCCTTT	60	98
Myod	ATCCGCTACATCGAAGGTCTG	CTCGACACAGCCGCACTCTTC	62	98
Myog	CCAGTGAATGCAACTCCCACAGC	AGACATATCCTCCACCGTGA	59	102
Col1a1	GAGCGGAGAGTACTGGATCG	GCTTCTTTTCCTTGGGGTTC	61.4	97
Col3a1	GCCCACAGCCTTCTACAC	CCAGGGTCACCATTTCTC	61.4	98
Myh3	TAGCCGGATGGTGGTCCAT	CCAACTTGAAACAAGGCAAAC	54	94
Myh2	ACTTTGGCACTACGGGGAAAC	CAGCAGCATTTCGATCAGCTC	61.4	96
hACAT1	CCTCGTGTGCGACAATGG	CACGTAGGAATCTTTCTGAC	61.4	98
Myh4	AAACCACCTCAGAGTTGTGGA	GTTCCGAAGGTTCCTGATTGC	61.4	97

Table S2. Primers for splicing assays

Gene	Forward primer	Reverse primer	Anneal-ing temp (°C)	Cycle
SYNE1	CTGCCCTCTGAGGATGAAGAAG	CGGCTATCATCCAGGGCTTTG	64	33x
Syne1	CTGCCCTCAGAAGATGAAGAAG	CGGCTGTCATCCAGGGCTTTG	64	33x
BIN1	AGAACCTCAATGATGTGCTGG	TCGTGGTTGACTCTGATCTCGG	58	32x
Bin1	TCAATGATGTCCTGGTCAGC	GCTCATGGTTCACTCTGATC	58	32x
